# Supplementary material for: Integrated plasma and vegetation proteomic characterization of infective endocarditis for early diagnosis and treatment
Source: Nat Commun. 2025 May 30;16:5052. doi: 10.1038/s41467-025-60184-8 (PMC12125238; doi:10.1038/s41467-025-60184-8)
Supplement: Supplementary file 1 — Supplementary Information [file 41467_2025_60184_MOESM1_ESM.pdf]

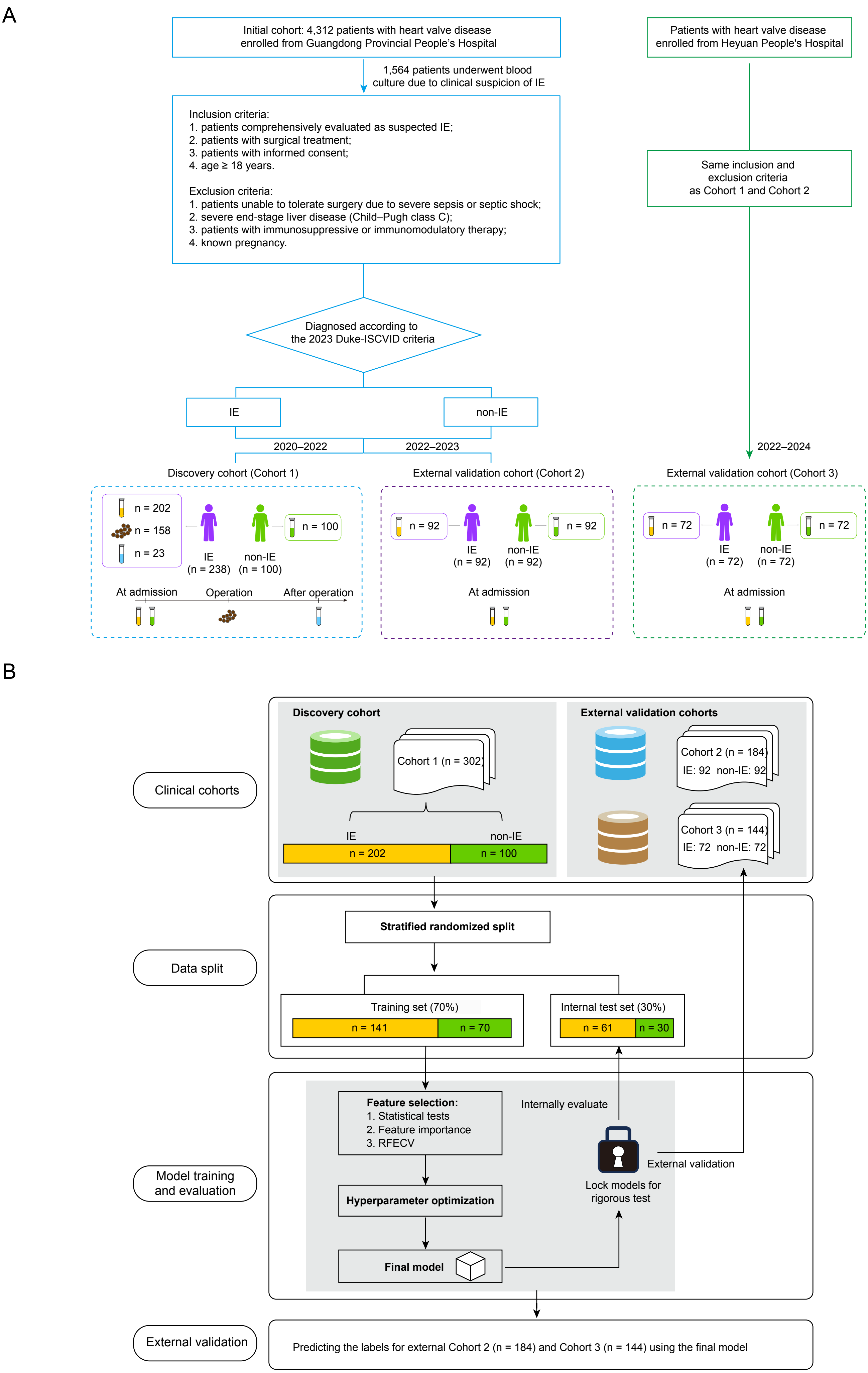
A
Initial cohort: 4,312 patients with heart valve disease enrolled from Guangdong Provincial People's Hospital
1,564 patients underwent blood culture due to clinical suspicion of IE
Inclusion criteria:
1. patients comprehensively evaluated as suspected IE;
2. patients with surgical treatment;
3. patients with informed consent;
4. age ≥ 18 years.
Exclusion criteria:
1. patients unable to tolerate surgery due to severe sepsis or septic shock;
2. severe end-stage liver disease (Child–Pugh class C);
3. patients with immunosuppressive or immunomodulatory therapy;
4. known pregnancy.
Diagnosed according to the 2023 Duke-ISCVID criteria
IE
non-IE
2020–2022
2022–2023
Patients with heart valve disease enrolled from Heyuan People's Hospital
Same inclusion and exclusion criteria as Cohort 1 and Cohort 2
2022–2024
Discovery cohort (Cohort 1)
n = 202
n = 158
n = 23
IE (n = 238)
non-IE (n = 100)
n = 100
At admission
Operation
After operation
External validation cohort (Cohort 2)
n = 92
IE (n = 92)
non-IE (n = 92)
n = 92
At admission
External validation cohort (Cohort 3)
n = 72
IE (n = 72)
non-IE (n = 72)
n = 72
At admission
B
Clinical cohorts
Discovery cohort
Cohort 1 (n = 302)
IE
non-IE
n = 202
n = 100
External validation cohorts
Cohort 2 (n = 184)
IE: 92 non-IE: 92
Cohort 3 (n = 144)
IE: 72 non-IE: 72
Data split
Stratified randomized split
Training set (70%)
n = 141
n = 70
Internal test set (30%)
n = 61
n = 30
Model training and evaluation
Feature selection:
1. Statistical tests
2. Feature importance
3. RFECV
Hyperparameter optimization
Final model
Internally evaluate
External validation
Lock models for rigorous test
External validation
Predicting the labels for external Cohort 2 (n = 184) and Cohort 3 (n = 144) using the final model

Supplementary Figure 1. Workflow of the patient enrollment process and model development. **A** Flowchart illustrating the patient inclusion and exclusion processes. **B** Schematic workflow for developing and evaluating the machine learning model.

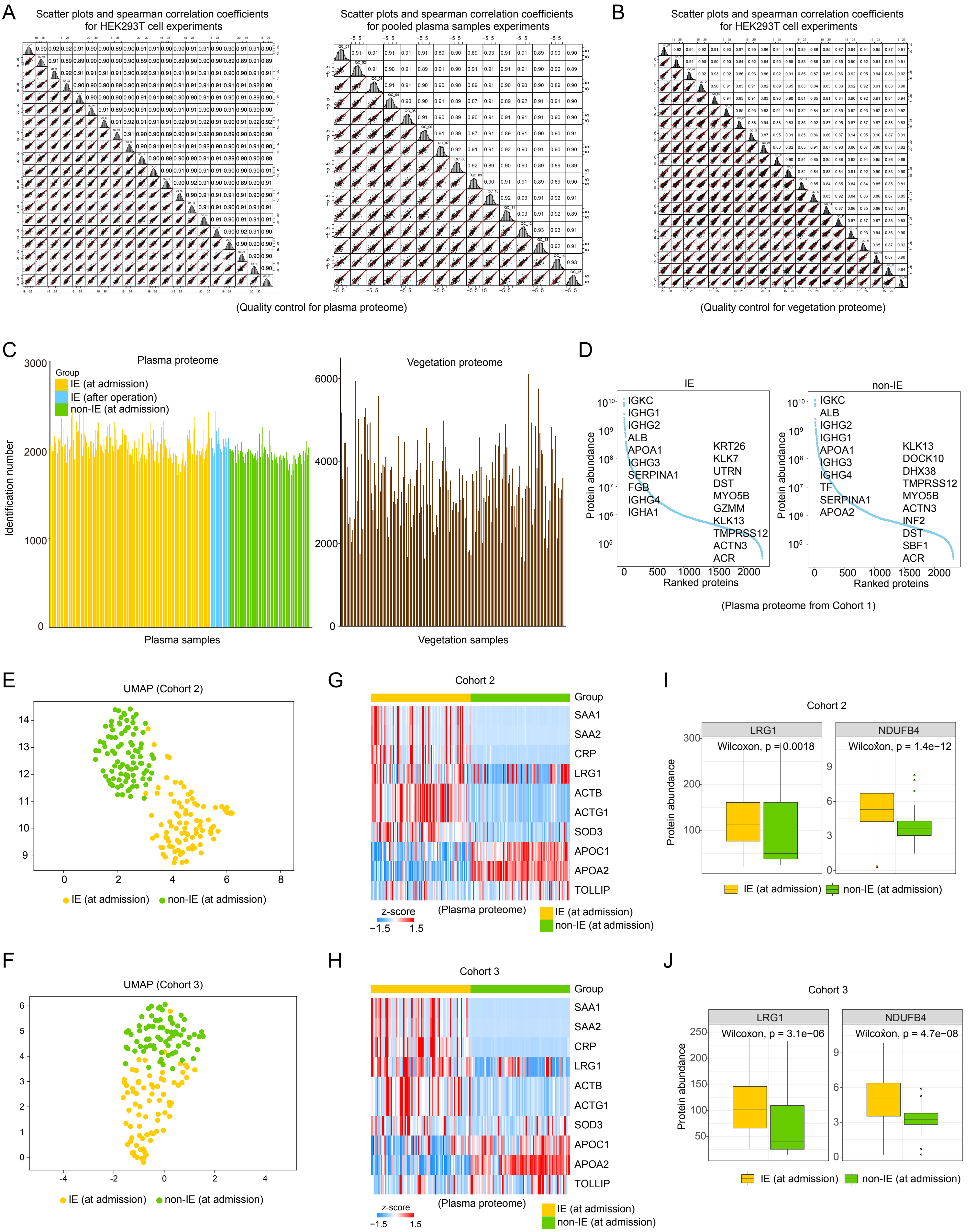

A
Scatter plots and spearman correlation coefficients
for HEK293T cell experiments
Scatter plots and spearman correlation coefficients
for pooled plasma samples experiments
(Quality control for plasma proteome)
B
Scatter plots and spearman correlation coefficients
for HEK293T cell experiments
(Quality control for vegetation proteome)
C
Plasma proteome
Group
IE (at admission)
IE (after operation)
non-IE (at admission)
Identification number
Plasma samples
Vegetation proteome
Vegetation samples
D
IE
IGKC
IGHG1
IGHG2
ALB
APOA1
IGHG3
SERPINA1
FGB
IGHG4
IGHA1
KRT26
KLK7
UTRN
DST
MYO5B
GZMM
KLK13
TMPRSS12
ACTN3
ACR
non-IE
IGKC
ALB
IGHG2
IGHG1
APOA1
IGHG3
IGHG4
TF
SERPINA1
APOA2
KLK13
DOCK10
DHX38
TMPRSS12
MYO5B
ACTN3
INF2
DST
SBF1
ACR
Protein abundance
Ranked proteins
(Plasma proteome from Cohort 1)
E
UMAP (Cohort 2)
IE (at admission)
non-IE (at admission)
G
Cohort 2
Group
SAA1
SAA2
CRP
LRG1
ACTB
ACTG1
SOD3
APOC1
APOA2
TOLLIP
z-score
(Plasma proteome)
IE (at admission)
non-IE (at admission)
I
Cohort 2
LRG1
Wilcoxon, p = 0.0018
NDUFB4
Wilcoxon, p = 1.4e−12
Protein abundance
IE (at admission)
non-IE (at admission)
F
UMAP (Cohort 3)
IE (at admission)
non-IE (at admission)
H
Cohort 3
Group
SAA1
SAA2
CRP
LRG1
ACTB
ACTG1
SOD3
APOC1
APOA2
TOLLIP
z-score
(Plasma proteome)
IE (at admission)
non-IE (at admission)
J
Cohort 3
LRG1
Wilcoxon, p = 3.1e−06
NDUFB4
Wilcoxon, p = 4.7e−08
Protein abundance
IE (at admission)
non-IE (at admission)

Supplementary Figure 2. Quality control and overview of the proteomic data. A, B Scatter plots illustrating the quality control for plasma and vegetation proteomes. For the plasma proteome (**A**), mass spectrometry (MS) stability was assessed using a tryptic digest of HEK293T cells (right, n = 20) and pooled plasma samples (left, n = 15), both with an average correlation coefficient of 0.90. For the vegetation proteome (**B**), a tryptic digest of HEK293T cells (n = 20) yielded an average correlation coefficient of 0.88. The top right panel of the scatter plot shows the two-sided Spearman's correlation coefficients, while the bottom left panel presents scatterplots. **C** Protein identification number across all plasma (n = 325) and vegetation (n = 158) samples in the discovery cohort (Cohort 1). **D** Ranking of proteins identified in the Cohort 1 based on their MS signals, spanning approximately 10 orders of magnitude. The top ten most and least abundant proteins are labeled. **E, F** UMAP of plasma samples in the external validation cohorts, Cohort 2 and Cohort 3. **G, H** Heatmap showing the expression profiles of the 10 proteins included in the developed diagnostic model in the Cohort 2 and Cohort 3. **I, J** Boxplots illustrating the abundance differences of LRG1 and NDUFB4 between IE and non-IE groups in the Cohort 2 (IE, n = 92; non-IE, n = 92) and Cohort 3 (IE, n = 72; non-IE, n = 72). The central line represents the median, the box indicates the interquartile range (IQR), and whiskers extend to 1.5×IQR, with outliers as individual points. The unpaired two-sided Wilcoxon rank-sum test was used for statistical test. Source data are provided as Source Data files.

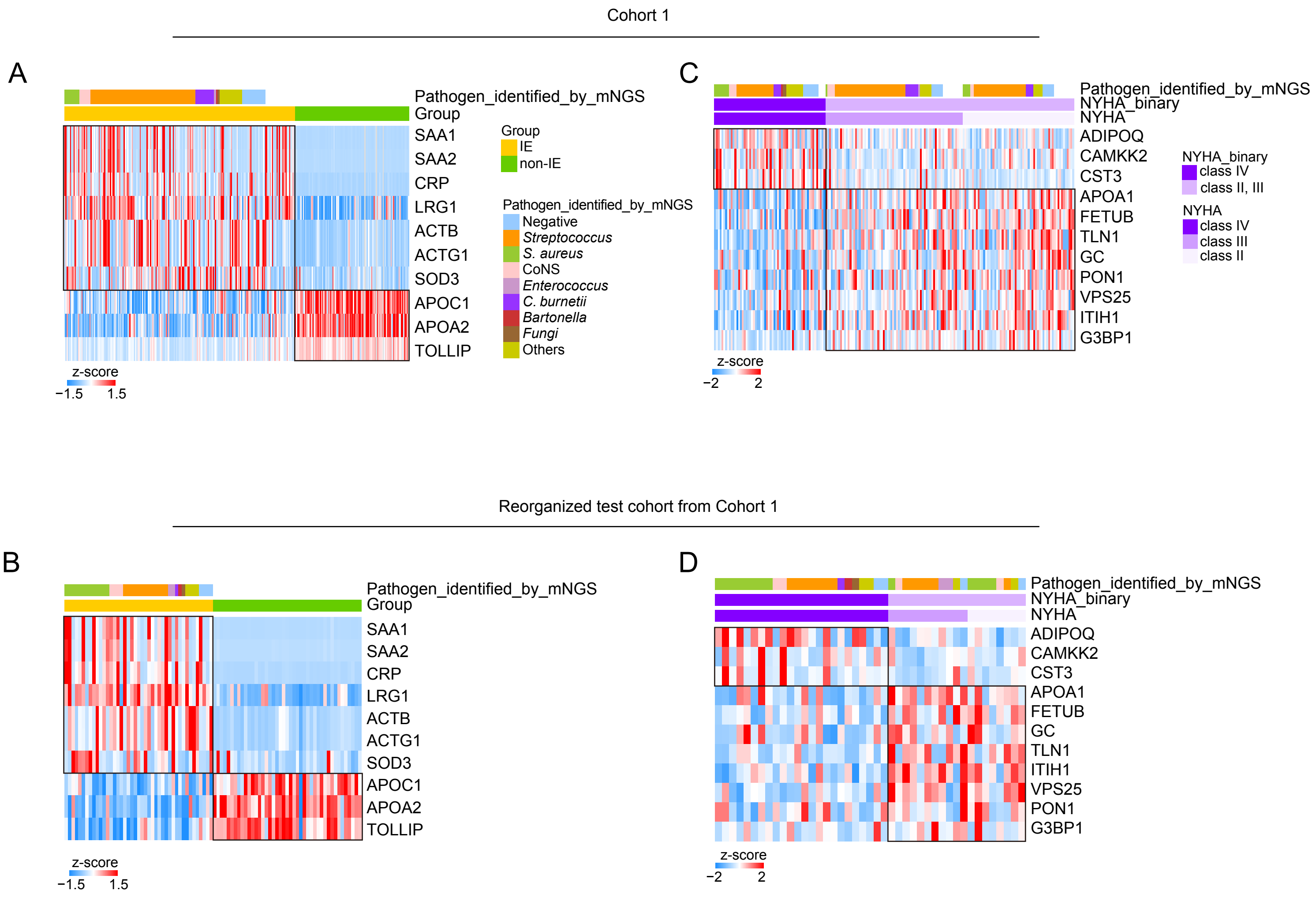
Cohort 1
A
Pathogen_identified_by_mNGS
Group
SAA1
SAA2
CRP
LRG1
ACTB
ACTG1
SOD3
APOC1
APOA2
TOLLIP
z-score
−1.5
1.5
Group
IE
non-IE
Pathogen_identified_by_mNGS
Negative
Streptococcus
S. aureus
CoNS
Enterococcus
C. burnetii
Bartonella
Fungi
Others
C
Pathogen_identified_by_mNGS
NYHA_binary
NYHA
ADIPOQ
CAMKK2
CST3
APOA1
FETUB
TLN1
GC
PON1
VPS25
ITIH1
G3BP1
z-score
−2
2
NYHA_binary
class IV
class II, III
NYHA
class IV
class III
class II
Reorganized test cohort from Cohort 1
B
Pathogen_identified_by_mNGS
Group
SAA1
SAA2
CRP
LRG1
ACTB
ACTG1
SOD3
APOC1
APOA2
TOLLIP
z-score
−1.5
1.5
D
Pathogen_identified_by_mNGS
NYHA_binary
NYHA
ADIPOQ
CAMKK2
CST3
APOA1
FETUB
GC
TLN1
ITIH1
VPS25
PON1
G3BP1
z-score
−2
2

Supplementary Figure 3. Preliminary evaluation of the efficacy of identified biomarkers utilizing a reorganized test cohort from the Cohort 1. A, B Heatmap showing the expression profiles of the 10 plasma proteins identified in the developed diagnostic model in the Cohort 1 and reorganized test cohort. **C, D** Heatmap depicting the expression profiles of the 11 plasma proteins identified in the prognostic model in the Cohort 1 and reorganized test cohort. For figures **A-D**, the annotation of pathogen identified by metagenomics next-generation sequencing (mNGS) for each sample is displayed above the heatmap, with blanks indicating missing records. Source data are provided as Source Data files.

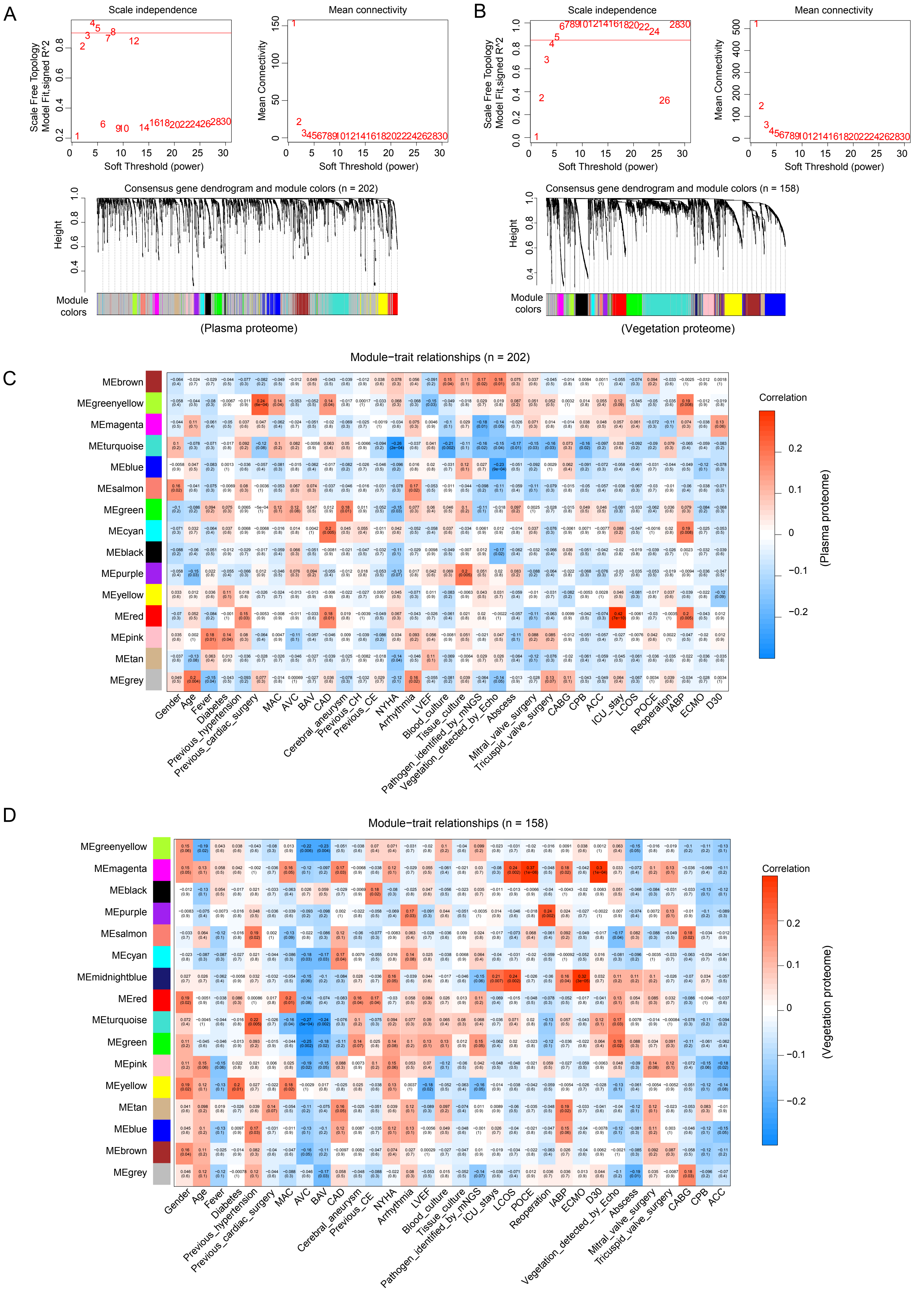

A
Scale independence
Scale Free Topology Model Fit,signed R^2
Soft Threshold (power)
Mean connectivity
Mean Connectivity
Soft Threshold (power)
B
Scale independence
Scale Free Topology Model Fit,signed R^2
Soft Threshold (power)
Mean connectivity
Mean Connectivity
Soft Threshold (power)
Consensus gene dendrogram and module colors (n = 202)
Height
Module colors
(Plasma proteome)
Consensus gene dendrogram and module colors (n = 158)
Height
Module colors
(Vegetation proteome)
C
Module−trait relationships (n = 202)
MEbrown
MEgreenyellow
MEmagenta
MEturquoise
MEblue
MEsalmon
MEgreen
MEcyan
MEblack
MEpurple
MEyellow
MEred
MEpink
MEtan
MEgrey
Gender
Age
Fever
Diabetes
Previous_hypertension
Previous_cardiac_surgery
MAC
AVC
BAV
CAD
Cerebral_aneurysm
Previous_CH
Previous_CE
NYHA
Arrhythmia
LVEF
Blood_culture
Tissue_culture
Pathogen_identified_by_mNGS
Vegetation_detected_by_Echo
Abscess
Mitral_valve_surgery
Tricuspid_valve_surgery
CABG
CPB
ACC
ICU_stay
LCOS
POCE
Reoperation
IABP
ECMO
D30
Correlation
(Plasma proteome)
D
Module−trait relationships (n = 158)
MEgreenyellow
MEmagenta
MEblack
MEpurple
MEsalmon
MEcyan
MEmidnightblue
MEred
MEturquoise
MEgreen
MEpink
MEyellow
MEtan
MEblue
MEbrown
MEgrey
Gender
Age
Fever
Diabetes
Previous_hypertension
Previous_cardiac_surgery
MAC
AVC
BAV
CAD
Cerebral_aneurysm
Previous_CE
NYHA
Arrhythmia
LVEF
Blood_culture
Tissue_culture
Pathogen_identified_by_mNGS
ICU_stays
LCOS
POCE
Reoperation
IABP
ECMO
D30
Vegetation_detected_by_Echo
Abscess
Mitral_valve_surgery
Tricuspid_valve_surgery
CABG
CPB
ACC
Correlation
(Vegetation proteome)

Supplementary Figure 4. Identification of IE-specific modules using WGCNA. A, B Automatic construction of protein networks and module identification for the plasma (**A**) and vegetation (**B**) proteomes, including network topology analysis for soft-thresholding power (top) and clustering dendrogram with module colors (bottom). **C, D** Heatmap of the correlation between module eigengenes and clinical traits in the plasma (**C**) and vegetation (**D**) proteomes. The two-sided Pearson's correlation (WGCNA-derived) was used for statistical test. Abbreviations: Previous CH, Previous cerebral hemorrhage; ACC: aortic cross clamp. Source data are provided as Source Data files.

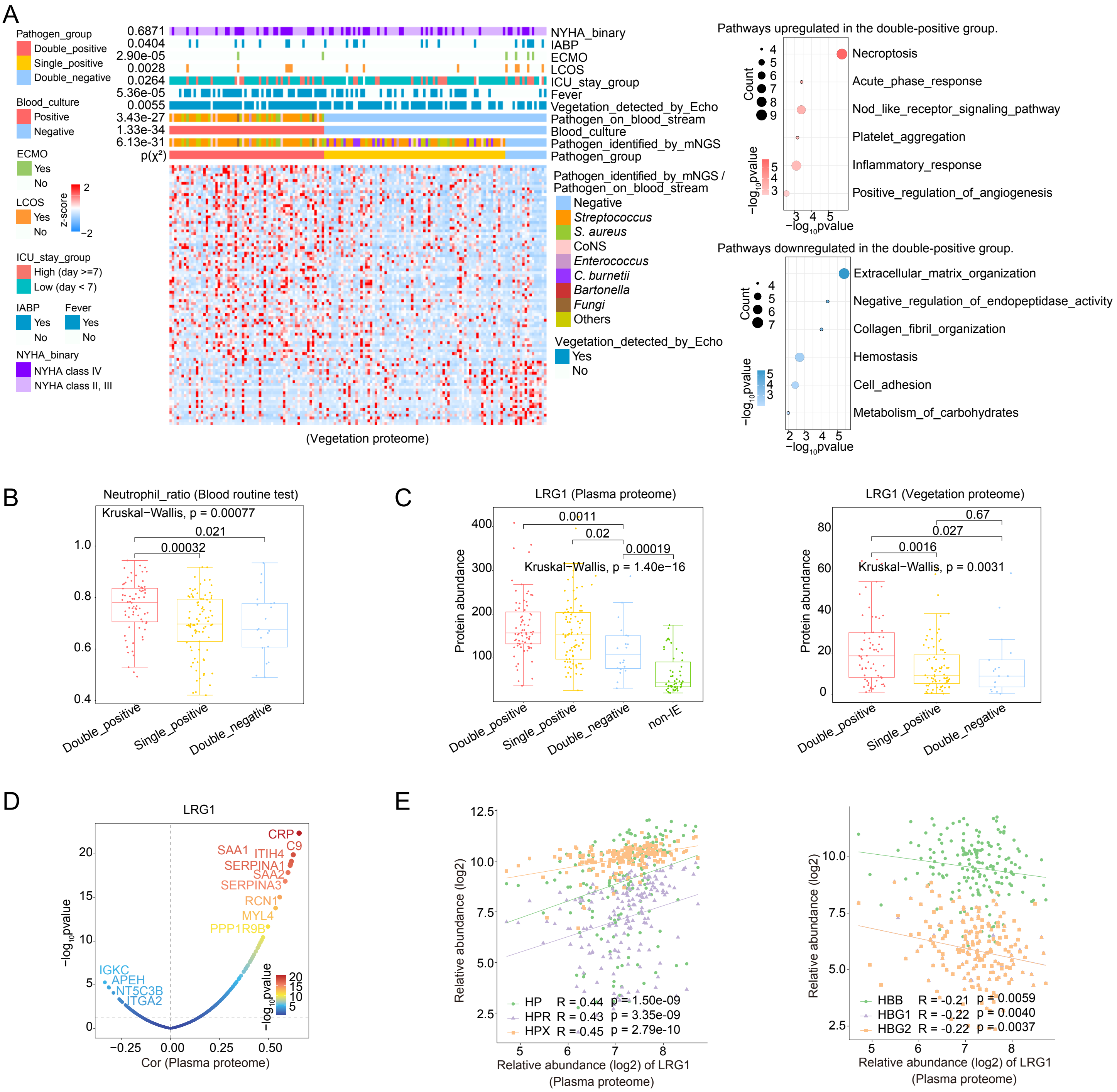
A
Pathogen_group
Double_positive
Single_positive
Double_negative
Blood_culture
Positive
Negative
ECMO
Yes
No
LCOS
Yes
No
z-score
2
−2
ICU_stay_group
High (day >=7)
Low (day < 7)
IABP
Yes
No
Fever
Yes
No
NYHA_binary
NYHA class IV
NYHA class II, III
0.6871 NYHA_binary
0.0404 IABP
2.90e-05 ECMO
0.0028 LCOS
0.0264 ICU_stay_group
5.36e-05 Fever
0.0055 Vegetation_detected_by_Echo
3.43e-27 Pathogen_on_blood_stream
1.33e-34 Blood_culture
6.13e-31 Pathogen_identified_by_mNGS
p(χ²) Pathogen_group
Pathogen_identified_by_mNGS / Pathogen_on_blood_stream
Negative
Streptococcus
S. aureus
CoNS
Enterococcus
C. burnetii
Bartonella
Fungi
Others
Vegetation_detected_by_Echo
Yes
No
(Vegetation proteome)
Pathways upregulated in the double-positive group.
Count
4
5
6
7
8
9
Necroptosis
Acute_phase_response
Nod_like_receptor_signaling_pathway
Platelet_aggregation
Inflammatory_response
Positive_regulation_of_angiogenesis
−log10pvalue
Pathways downregulated in the double-positive group.
Extracellular_matrix_organization
Negative_regulation_of_endopeptidase_activity
Collagen_fibril_organization
Hemostasis
Cell_adhesion
Metabolism_of_carbohydrates
B
Neutrophil_ratio (Blood routine test)
Kruskal−Wallis, p = 0.00077
0.021
0.00032
Double_positive
Single_positive
Double_negative
C
LRG1 (Plasma proteome)
Protein abundance
0.0011
0.02
0.00019
Kruskal−Wallis, p = 1.40e−16
Double_positive
Single_positive
Double_negative
non-IE
LRG1 (Vegetation proteome)
0.67
0.027
0.0016
Kruskal−Wallis, p = 0.0031
D
LRG1
CRP
C9
SAA1
ITIH4
SERPINA1
SAA2
SERPINA3
RCN1
MYL4
PPP1R9B
IGKC
APEH
NT5C3B
ITGA2
−log10pvalue
Cor (Plasma proteome)
E
Relative abundance (log2)
HP R = 0.44 p = 1.50e-09
HPR R = 0.43 p = 3.35e-09
HPX R = 0.45 p = 2.79e-10
Relative abundance (log2) of LRG1 (Plasma proteome)
HBB R = -0.21 p = 0.0059
HBG1 R = -0.22 p = 0.0040
HBG2 R = -0.22 p = 0.0037
Relative abundance (log2) of LRG1 (Plasma proteome)

Supplementary Figure 5. Proteomic alterations associated with infection severity in IE. A Heatmap and bubble chart showing the two panels of vegetation proteins with gradually increasing (top) or decreasing (bottom) abundance from double-positive to single-positive, and then to double-negative group, along with pathway enrichment. Pearson's two-sided χ^2 test was used to evaluate the associations between the pathogen group and 10 variables. **B, C** Boxplots depicting the level of neutrophil ratio (**B**) and LRG1 (**C**) across double-positive (**B** and **C** left, n = 71; **C** right, n = 64), single-positive (**B** and **C** left, n = 85; **C** right, n = 75), and double-negative (**B** and **C** left, n = 20; **C** right, n = 17), and non-IE (**C** left, n = 50) groups. **D** Scatterplot showing the correlations of LRG1 with other proteins in the plasma proteome. **E** Scatterplot showing the correlations between LRG1 and the proteins of haptoglobin (HP and HPR), hemopexin (HPX), and hemoglobin (HBB, HBG1, and HBG2) in the plasma proteome, respectively. Each dot represents a patient. For boxplots **B** and **C**, the central line represents the median, the box indicates the IQR, and whiskers extend to 1.5×IQR, with outliers as individual points. The unpaired two-sided Wilcoxon rank-sum test and Kruskal–Wallis test were used for statistical test. For figures **D** and **E**, the two-sided Spearman's correlation was used, with p-values adjusted for FDR. Source data are provided as Source Data files.

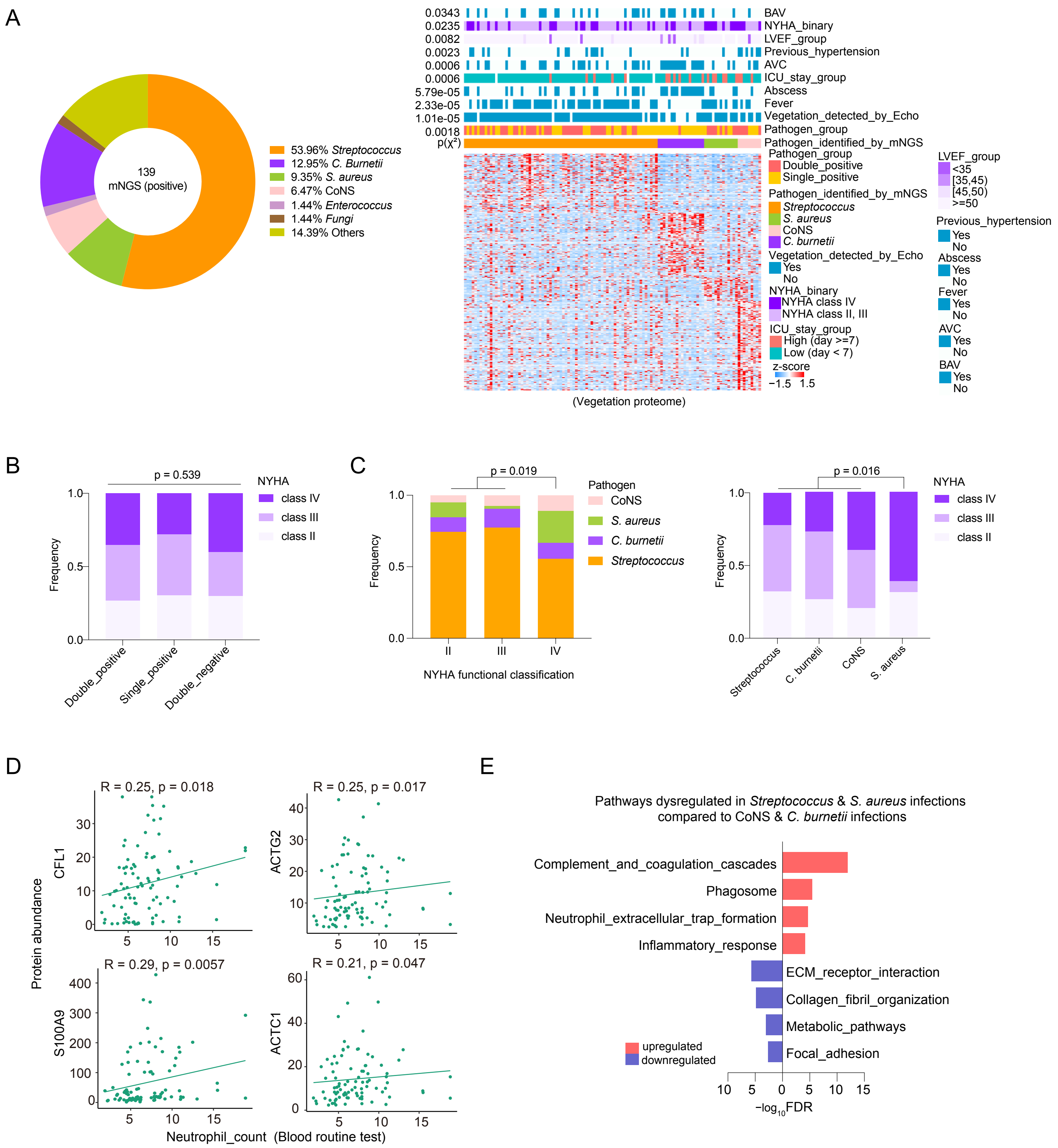

A
139
mNGS (positive)
53.96% Streptococcus
12.95% C. Burnetii
9.35% S. aureus
6.47% CoNS
1.44% Enterococcus
1.44% Fungi
14.39% Others
0.0343 BAV
0.0235 NYHA_binary
0.0082 LVEF_group
0.0023 Previous_hypertension
0.0006 AVC
0.0006 ICU_stay_group
5.79e-05 Abscess
2.33e-05 Fever
1.01e-05 Vegetation_detected_by_Echo
0.0018 Pathogen_group
p(χ²) Pathogen_identified_by_mNGS
Pathogen_group
Double_positive
Single_positive
Pathogen_identified_by_mNGS
Streptococcus
S. aureus
CoNS
C. burnetii
Vegetation_detected_by_Echo
Yes
No
NYHA_binary
NYHA class IV
NYHA class II, III
ICU_stay_group
High (day >=7)
Low (day < 7)
z-score
−1.5 1.5
LVEF_group
<35
[35,45)
[45,50)
>=50
Previous_hypertension
Yes
No
Abscess
Yes
No
Fever
Yes
No
AVC
Yes
No
BAV
Yes
No
(Vegetation proteome)
B
p = 0.539
NYHA
class IV
class III
class II
Frequency
Double_positive
Single_positive
Double_negative
C
p = 0.019
Pathogen
CoNS
S. aureus
C. burnetii
Streptococcus
Frequency
II
III
IV
NYHA functional classification
p = 0.016
NYHA
class IV
class III
class II
Frequency
Streptococcus
C. burnetii
CoNS
S. aureus
D
R = 0.25, p = 0.018
CFL1
R = 0.25, p = 0.017
ACTG2
R = 0.29, p = 0.0057
S100A9
R = 0.21, p = 0.047
ACTC1
Protein abundance
Neutrophil_count (Blood routine test)
E
Pathways dysregulated in Streptococcus & S. aureus infections compared to CoNS & C. burnetii infections
Complement_and_coagulation_cascades
Phagosome
Neutrophil_extracellular_trap_formation
Inflammatory_response
ECM_receptor_interaction
Collagen_fibril_organization
Metabolic_pathways
Focal_adhesion
upregulated
downregulated
−log10FDR

Supplementary Figure 6. Proteomic alterations associated with bacterial species in IE. A Doughnut chart showing pathogen proportions among 139 IE patients with positive mNGS results from Cohort 1 (vegetation sample cohort). Heatmap showing differential vegetation proteomic profiles across *Streptococcus*, *C. burnetii*, *S. aureus*, and CoNS groups. **B** Stacked bar plots illustrating the patient proportions with various NYHA class among the double-positive, single-positive, and double-negative groups. **C** Stacked bar plots comparing the patient proportions of pathogen infections between NYHA class IV and NYHA class II, III groups (left), as well as the patient proportions of NYHA class between *S. aureus* infection and the other three pathogen infections (right). **D** Scatter plots showing the two-sided Spearman's correlation between the abundance of actin-related proteins including CFL1, ACTG2, S100A9, and ACTC1 (y-axis) and neutrophil count (x-axis), respectively. **E** Bar chart showing the dysregulated pathways in *Streptococcus* & *S. aureus* infections compared to *C. burnetii* & CoNS infections in the vegetation proteome. For figures **A-C**, Pearson's two-sided χ^2 test was used for statistical rest. Source data are provided as Source Data files.
