# Supplementary material for: Integrated plasma and vegetation proteomic characterization of infective endocarditis for early diagnosis and treatment
Source: Nat Commun. 2025 May 30;16:5052. doi: 10.1038/s41467-025-60184-8 (PMC12125238; doi:10.1038/s41467-025-60184-8)
Supplement: Supplementary file 2 — Description of Additional Supplementary Files [file 41467_2025_60184_MOESM2_ESM.docx]

File Name: Supplementary Data 1
Description:

Baseline characteristics of participants included in the study, summarizing the clinical traits across Cohorts 1, 2, and 3. The three-line table within the file details the clinical characteristics of the cohorts, with continuous variables shown as mean (standard deviation) and categorical variables as number (percentage).
