# Supplementary material for: Integrated plasma and vegetation proteomic characterization of infective endocarditis for early diagnosis and treatment
Source: Nat Commun. 2025 May 30;16:5052. doi: 10.1038/s41467-025-60184-8 (PMC12125238; doi:10.1038/s41467-025-60184-8)
Supplement: Supplementary file 4 — Reporting Summary [file 41467_2025_60184_MOESM4_ESM.pdf]

nature portfolio

Corresponding author(s): Chen Ding, Xuhua Jian, Bing Gu

Last updated by author(s): Apr 23, 2025

Reporting Summary

Nature Portfolio wishes to improve the reproducibility of the work that we publish. This form provides structure for consistency and transparency in reporting. For further information on Nature Portfolio policies, see our Editorial Policies and the Editorial Policy Checklist.

Statistics

For all statistical analyses, confirm that the following items are present in the figure legend, table legend, main text, or Methods section.

n/a	Confirmed	
☐	☒	The exact sample size (*n*) for each experimental group/condition, given as a discrete number and unit of measurement
☐	☒	A statement on whether measurements were taken from distinct samples or whether the same sample was measured repeatedly
☐	☒	The statistical test(s) used AND whether they are one- or two-sided<br>*Only common tests should be described solely by name; describe more complex techniques in the Methods section.*
☒	☐	A description of all covariates tested
☐	☒	A description of any assumptions or corrections, such as tests of normality and adjustment for multiple comparisons
☐	☒	A full description of the statistical parameters including central tendency (e.g. means) or other basic estimates (e.g. regression coefficient) AND variation (e.g. standard deviation) or associated estimates of uncertainty (e.g. confidence intervals)
☐	☒	For null hypothesis testing, the test statistic (e.g. *F*, *t*, *r*) with confidence intervals, effect sizes, degrees of freedom and *P* value noted<br>*Give P values as exact values whenever suitable.*
☒	☐	For Bayesian analysis, information on the choice of priors and Markov chain Monte Carlo settings
☐	☒	For hierarchical and complex designs, identification of the appropriate level for tests and full reporting of outcomes
☐	☒	Estimates of effect sizes (e.g. Cohen's *d*, Pearson's *r*), indicating how they were calculated

Our web collection on statistics for biologists contains articles on many of the points above.

Software and code

Policy information about availability of computer code

Data collection	The plasma proteomic raw data were performed by Q Exactive HF-X Mass Spectrometer, Thermo Fisher Scientific. The vegetation proteomic raw data were performed by Orbitrap Fusion Lumos Mass Spectrometer, Thermo Fisher Scientific.
Data analysis	All mass spectrometry raw files from plasma (DIA mode) and vegetation (DDA mode) samples were processed through the Firmiana cloud platform. The DDA data were searched using the Mascot 2.4 search engine configured in Firmiana. The DIA data analysis was conducted through two complementary approaches: (1) database searching with FragPipe (v12.1) utilizing MSFragger (v2.2), and (2) spectral library-based analysis with DIA-NN (v1.8.1), both implemented within the Firmiana environment.<br><br>The data analysis was performed in the R (v4.1.2) and python (v3.9.7) environments with open-source libraries. R Packages: ggplot2 (3.4.0), pheatmap (1.0.12), dplyr (1.0.10), Hmisc (v5.1-1),WGCNA (v1.72), GSVA (v1.50.2). Python Packages: numpy (v1.23.5), pandas (v1.2.3), matplotlib (3.9.1), scikit-learn (v1.1.3), seaborn (0.13.2), xgboost (v1.5.0), catboost (v1.1.1), lightgbm (v3.2.1). Pathway enrichment analysis was conducted with online tools DAVID (https://david.ncifcrf.gov/tools.jsp) and ConsensusPathDB (http://cpdb.molgen.mpg.de/). Immunofluorescence staining was analysed using SlideViewer (v2.5) software.

For manuscripts utilizing custom algorithms or software that are central to the research but not yet described in published literature, software must be made available to editors and reviewers. We strongly encourage code deposition in a community repository (e.g. GitHub). See the Nature Portfolio guidelines for submitting code & software for further information.

Data

Policy information about availability of data

All manuscripts must include a data availability statement. This statement should provide the following information, where applicable:

- Accession codes, unique identifiers, or web links for publicly available datasets
- A description of any restrictions on data availability
- For clinical datasets or third party data, please ensure that the statement adheres to our policy

The raw mass spectrometry proteomic data generated in this study have been deposited to the ProteomeXchange Consortium (https://proteomecentral.proteomexchange.org) via the iProX partner repository with the dataset identifier PXD062668. All software tools and publicly available resources used in this study are detailed in the Methods section. Additional processed data supporting the findings are available in the Supplementary Information or Source Data files. Source data are provided with this paper.

Research involving human participants, their data, or biological material

Policy information about studies with human participants or human data. See also policy information about sex, gender (identity/presentation), and sexual orientation and race, ethnicity and racism.

Reporting on sex and gender	This study does not pertain to issues of sex and gender.
Reporting on race, ethnicity, or other socially relevant groupings	The participants in our study are all Chinese people and they were not classified into subgroups based on their race, ethnicity, or other socially relevant grouping.
Population characteristics	After screening with predefined inclusion and exclusion criteria, eligible participants (all adults ≥18 years) were randomized into three cohorts with comparable demographic profiles: The median age was approximately 50 years across all cohorts (Cohort 1: 50.7±15.2; Cohort 2: 48.6±15.0; Cohort 3: 51.7±16.7 years, mean±SD), with male predominance (Cohort 1: 71.4%; Cohort 2: 59.8%; Cohort 3: 72.2%) reflecting the disease's epidemiological characteristics.
Recruitment	Initially, we prospectively collected a preliminary cohort of 4,312 patients with heart valve disease from Guangdong Provincial People's Hospital. Among them, a total of 1,564 patients initially underwent blood culture due to clinical suspicion of IE. Both IE patients and non-IE individuals (control group) included in the discovery cohort (Cohort 1) and the external validation cohort (Cohort 2) of this study were selected from the above initial cohort. All IE patients and non-IE individuals included in our study were diagnosed according to the 2023 Duke-ISCVID Criteria. To ensure a homogeneous study population and minimize confounding factors, we applied strict inclusion and exclusion criteria. The inclusion criteria were as follows: (1) definite IE and non-IE individuals diagnosed according to the 2023 Duke-ISCVID Criteria; (2) patients with informed consent; (3) age ≥ 18 years; (4) patients with surgical treatment; and (5) patients with definitive microbiological assessment results, including blood culture, vegetation culture, and/or pathogen identification through mNGS of blood or vegetation samples. The exclusion criteria included: (1) patients unable to tolerate surgery due to severe sepsis or septic shock; (2) severe end-stage liver disease (Child-Pugh class C); (3) known pregnancy; and (4) patients with immunosuppressive or immunomodulatory therapy. Ultimately, we included a total of 522 individuals from the initial cohort, comprising a discovery cohort (Cohort 1) with 238 IE patients and 100 non-IE individuals, and an external validation cohort (Cohort 2) with 92 IE patients and 92 non-IE individuals. Furthermore, following the above-mentioned inclusion and exclusion criteria, we additionally collected an external validation cohort (Cohort 3) consisting of 72 IE patients and 72 non-IE individuals from an external center, Heyuan People's Hospital.
Ethics oversight	The Research Ethics Committee of Guangdong Provincial People's Hospital, Guangdong Academy of Medical Sciences approved this study (Approval No. KY-N-2022-003-03), and written informed consent was obtained from all participants before enrollment. All procedures were conducted in accordance with the ethical principles outlined in the Declaration of Helsinki.

Note that full information on the approval of the study protocol must also be provided in the manuscript.

Field-specific reporting

Please select the one below that is the best fit for your research. If you are not sure, read the appropriate sections before making your selection.

☒ Life sciences ☐ Behavioural & social sciences ☐ Ecological, evolutionary & environmental sciences

For a reference copy of the document with all sections, see nature.com/documents/nr-reporting-summary-flat.pdf

Life sciences study design

All studies must disclose on these points even when the disclosure is negative.

Sample size	In this study, high-resolution MS-based plasma and vegetation proteomic analyses were performed on a total of 402 IE patients and 264 non-IE individuals from the discovery cohort (Cohort 1) and two external validation cohorts (Cohort 2 and Cohort 3).
Data exclusions	The exclusion criteria included: (1) patients unable to tolerate surgery due to severe sepsis or septic shock; (2) severe end-stage liver disease (Child-Pugh class C); (3) known pregnancy; and (4) patients with immunosuppressive or immunomodulatory therapy.

Replication | We implemented a rigorous quality control and validation framework to ensure data reliability. First, mass spectrometer performance was monitored through replicate analyses of 293T cell lysates. Second, each experimental batch included a pooled reference sample containing equal aliquots of all peptide samples, which served for both inter-batch quantitative accuracy assessment. Furthermore, our findings were further validated in two independent external cohorts. Statistical analyses were performed with more than three biological replicates per group to ensure adequate statistical power.

Randomization | In this study, randomization was applied to both the collection of clinical samples and the acquisition of proteomic data. Clinical samples were randomized after following predefined inclusion and exclusion criteria, ensuring unbiased selection. The proteomic data acquisition was also fully randomized, minimizing potential biases in the outcomes.

Blinding | In this study, rigorous blinding procedures were implemented throughout the experimental workflow. Clinical samples were collected by unblinded clinical staff according to predefined inclusion and exclusion criteria. However, laboratory investigators measuring protein expression were blinded to patient clinical information during data collection and analysis. Similarly, all bioinformatics analyses were conducted under blinded conditions.

Reporting for specific materials, systems and methods

We require information from authors about some types of materials, experimental systems and methods used in many studies. Here, indicate whether each material, system or method listed is relevant to your study. If you are not sure if a list item applies to your research, read the appropriate section before selecting a response.

Materials & experimental systems

n/a	Involved in the study
☐	☒ Antibodies
☐	☒ Eukaryotic cell lines
☒	☐ Palaeontology and archaeology
☒	☐ Animals and other organisms
☒	☐ Clinical data
☒	☐ Dual use research of concern
☒	☐ Plants

Methods

n/a	Involved in the study
☒	☐ ChIP-seq
☒	☐ Flow cytometry
☒	☐ MRI-based neuroimaging

Antibodies

Antibodies used | Anti-CitH3 (1:500, ab281584, Abcam);
Anti-MPO (1:3000, ab208670, Abcam);

Validation | Anti-CitH3 (1:500, ab281584, Abcam) validated for immunofluorescence staining by manufacturer [https://www.abcam.com/products/primary-antibodies/histone-h3-citrulline-r2--r8--r17-antibody-rm1001-ab281584.html];
Anti-MPO (1:3000, ab208670, Abcam) validated for immunohistochemistry by manufacturer [https://www.abcam.com/products/primary-antibodies/myeloperoxidase-antibody-epr20257-ab208670.html].

Eukaryotic cell lines

Policy information about cell lines and Sex and Gender in Research

Cell line source(s) | The HEK293T cell line (ATCC CRL-11268, RRID: CVCL_QW54) was obtained from the Chinese Academy of Sciences.

Authentication | All cell lines were routinely tested for mycoplasma contamination and authenticated by Short Tandem repeat (STR) profiling.

Mycoplasma contamination | All cell lines tested negative for mycoplasma contamination.

Commonly misidentified lines (See ICLAC register) | No commonly misidentified cell lines were used.

Plants

Seed stocks	No plants were used.
Novel plant genotypes	No plants were used.
Authentication	No plants were used.
